# Recruitment of men to a multi-centre diabetes prevention trial: an evaluation of traditional and online promotional strategies

**DOI:** 10.1186/s13063-019-3485-2

**Published:** 2019-06-19

**Authors:** Karen Bracken, Wendy Hague, Anthony Keech, Ann Conway, David J. Handelsman, Mathis Grossmann, David Jesudason, Bronwyn Stuckey, Bu B. Yeap, Warrick Inder, Carolyn Allan, Robert McLachlan, Kristy P. Robledo, Gary Wittert

**Affiliations:** 1 NHMRC Clinical Trials Centre, University of Sydney, Sydney, NSW Australia; 2 Anzac Research Institute, and Andrology Department, Concord Hospital, Sydney, NSW Australia; 3 Department of Medicine, the University of Melbourne, and Department of Endocrinology, Austin Health, Melbourne, VIC Australia; 4 Queen Elizabeth Hospital, Adelaide, SA Australia; 5 Department of Endocrinology and Diabetes, Keogh Institute of Medical Research, Sir Charles Gairdner Hospital and Medical School, University of Western Australia, Perth, WA Australia; 6 Department of Endocrinology and Diabetes, Medical School, University of Western Australia and Fiona Stanley Hospital, Perth, WA Australia; 7 Princess Alexandra Hospital, Brisbane, QLD Australia; 8 Department of Clinical Research, Hudson Institute of Medical Research, Melbourne, VIC Australia; 9 Freemasons Foundation Centre for Men’s Health, School of Medicine, University of Adelaide, Adelaide, SA Australia

**Keywords:** Participant recruitment, Recruitment strategies, Men’s health, Randomised controlled trials, Diabetes prevention, Advertising, Social media

## Abstract

**Background:**

Effective interventions are required to prevent the current rapid increase in the prevalence of Type 2 diabetes. Clinical trials of large-scale interventions to prevent Type 2 diabetes are essential but recruitment is challenging and expensive, and there are limited data regarding the most cost-effective and efficient approaches to recruitment. This paper aims to evaluate the cost and effectiveness of a range of promotional strategies used to recruit men to a large Type 2 diabetes prevention trial.

**Methods:**

An observational study was conducted nested within the Testosterone for the Prevention of Type 2 Diabetes (T4DM) study, a large, multi-centre randomised controlled trial (RCT) of testosterone treatment for the prevention of Type 2 diabetes in men aged 50–74 years at high risk of developing diabetes. Study participation was promoted via mainstream media—television, newspaper and radio; direct marketing using mass mail-outs, publicly displayed posters and attendance at local events; digital platforms, including Facebook and Google; and online promotions by community organisations and businesses. For each strategy, the resulting number of participants and the direct cost involved were recorded. The staff effort required for each strategy was estimated based on feedback from staff.

**Results:**

Of 19,022 men screened for the study, 1007 (5%) were enrolled. The most effective recruitment strategies were targeted radio advertising (accounting for 42% of participants), television news coverage (20%) and mass mail-outs (17%). Other strategies, including radio news, publicly displayed posters, attendance at local events, newspaper advertising, online promotions and Google and Facebook advertising, each accounted for no more than 4% of enrolled participants. Recruitment promotions cost an average of AU$594 per randomised participant. The most cost-effective paid strategy was mass mail-outs by a government health agency (AU$745 per participant). Other paid strategies were more expensive: mail-out by general practitioners (GPs) (AU$1104 per participant), radio advertising (AU$1081) and newspaper advertising (AU$1941).

**Conclusion:**

Radio advertising, television news coverage and mass mail-outs by a government health agency were the most effective recruitment strategies. Close monitoring of recruitment outcomes and ongoing enhancement of recruitment activities played a central role in recruitment to this RCT.

**Trial registration:**

ANZCTR, ID: ACTRN12612000287831. Registered on 12 March 2012.

**Electronic supplementary material:**

The online version of this article (10.1186/s13063-019-3485-2) contains supplementary material, which is available to authorized users.

## Background

Worldwide, an estimated 1 in 11 adults has diabetes, and Type 2 diabetes accounts for 90% of these cases [1]. Research to identify effective interventions to prevent diabetes is urgently needed to address this global problem. However, recruitment to disease prevention trials, including diabetes prevention trials, can be challenging. Firstly, since participants in disease prevention trials tend to be healthy and asymptomatic, clinicians may not be able to identify eligible patients through their clinics [2]. Secondly, potential participants may not perceive benefit in participating in disease prevention research, particularly if they do not believe that they are at risk of the disease [3, 4]. Lack of perceived benefit may contribute to lower rates of consent, requiring larger numbers of people to be screened [5]. Thirdly, screening numbers must be large in prevention trials if a modest effect size is hypothesised to ensure adequate power.

To overcome the challenge of recruiting sufficient numbers of participants, previous diabetes prevention trials have reported promoting study participation through: media coverage [6–9], advertising [7–11], mass mailings [7, 9, 12], referrals from physicians or clinics [6, 7, 9, 10, 12], community-based initiatives [6, 7, 10, 12, 13] and public screening events [7–9, 12]. Evaluations of these same recruitment strategies have also been reported in other research areas, including lifestyle improvement interventions [14], smoking cessation [15] and treatment of benign prostatic hyperplasia [16, 17]. While the existing literature provides useful recruitment guidance, papers have often lacked sufficient detail on how strategies were implemented and delivered, and how much they cost [18], making replication difficult [19]. Evaluations of approaches to promote randomised controlled trial (RCT) participation to members of the public is an area of research need, identified as one of the top ten areas for recruitment methodology research in a recent priority-setting study [20].

Recently, online recruitment through Facebook and Google advertising has been reported to be both affordable and effective in recruiting participants to survey research [21] and trials of short duration involving web-based interventions [22, 23]. Online advertising has some advantages over more traditional promotional strategies as it is faster to implement, easier to monitor, has lower start-up costs and can potentially reach larger numbers of people quickly [24]. However, to date, most evaluations of online strategies to recruit to RCTs have focussed on recruiting younger people [24–26]. Furthermore, evidence on the effectiveness of online strategies in the recruitment of men is mixed. Two studies found online promotions less effective in recruiting men compared to women [27, 28], but one found no significant difference in gender balance between online and traditional approaches [24]. More evidence is needed to assess whether online recruitment strategies are effective in recruiting middle-aged and older men to RCTs [29].

The aim of this study was to describe and evaluate the strategies used to promote recruitment to the T4DM diabetes prevention RCT.

## Methods

### Setting

This observational study of recruitment strategies was set within the Testosterone for the Prevention of Type 2 Diabetes (T4DM) trial (ACTRN12612000287831). The design of the T4DM trial has been published separately [30]. Briefly, T4DM is a large, multi-centre, phase-III, double-blind, placebo-controlled, 2-year trial of testosterone therapy combined with a lifestyle intervention (Weight Watchers®) compared to the lifestyle intervention alone for the prevention of Type 2 diabetes. The trial is running through six centres in Australian capital cities and recruitment occurred from January 2013 to February 2017. The trial enrolled men aged 50–74 years who were overweight or obese (≥ 95 cm waist circumference), had pre-diabetes or newly diagnosed Type 2 diabetes, and testosterone level ≤ 14.0 nmol/L. The trial is ongoing and follow-up is due to be completed in May 2019.

The T4DM trial design presented a number of recruitment challenges. Firstly, very few participants could be referred to the study by investigators at the participating centres since prospective participants were unlikely to be under the care of an endocrinologist. We therefore planned to seek prospective participants directly from the community, predicting that only one in four men screened in this non-targeted way would be eligible based on the entry criteria of elevated blood glucose and low serum testosterone. Secondly, we predicted that the placebo-controlled and injectable nature of the study treatment, as well as its 2-year duration, might limit the number of men willing to participate. Taking these two factors into account, we estimated that approximately 20,000 men would need to be screened in order to reach the final recruitment target of 1000 participants.

### Screening and enrolment process

Men who heard about the study through the promotional strategies to be described in this paper were invited to complete a pre-screening questionnaire online (on the T4DM study website, www.diabetesprevention.org.au), or over the telephone (by calling the central coordinating centre). Men who were eligible on the pre-screening questionnaire were emailed or posted a pre-screening patient information and consent form, instructions and a request form to attend for screening blood tests to be conducted at one of a large number of contracted pathology collection centres from one national commercial pathology provider company. Participants who were eligible on the screening blood tests were then contacted by their preferred centre to arrange a screening clinic visit. Eligible and consenting participants were enrolled and randomised at the clinic.

### Recruitment oversight and planning

During the recruitment phase, the Steering Committee met monthly by telephone to oversee the recruitment plan and monitor the ongoing performance of recruitment strategies. The Human Review Ethics Committees approved the study’s recruitment strategies and promotional material. Where promotional activities involved real-time communication with the public; for example, through Facebook posts, the approach to be taken with these communications, and the subject matter to be covered, received ethical review and approval.

Development of the recruitment plan involved four key considerations based on marketing principles [31]: selection of the target audience, definition of the call to action, design of the promotional material and selection of promotional strategies to be used (Table 1).

**Table 1:** Recruitment plan formulation

Planning considerations	Details
Target audience	Men aged 50–74 years who were overweight or obese and living in a capital city with a participating study centre. No further restrictions were placed as other eligibility criteria were to be assessed during the screening process
Call to action	Prospective participants were invited to visit study website or call a central information line to learn more about the study and complete the pre-screening questionnaire
Content of promotional material	Content decisions were guided by qualitative research in men’s health communication preferences [32], pro-bono advice from marketing professionals, and pre-testing and ongoing feedback from study participants Communication style: • Frank, humorous and empathetic message [32] • Simple, informal and easy-to-remember language Key components of the message: 1. Identification of the problem: men aged 50–74 years and overweight/obese are at risk of diabetes, weight gain and urinary and sexual problems 2. Positioning of the study as a solution: the Testosterone for the Prevention of Type 2 Diabetes (T4DM) study can support men to lose excess weight and address related health issues 3. Call to action: invitation to join the study and instructions on how to join
Promotional strategies/platforms	Promising promotional strategies were identified by review of the published literature, discussion with the study’s industry partners, brainstorming by the Steering Committee, suggestions from study participants and pro-bono advice from marketing professionals. Strategies were first tested for a short period of time, and if they appeared effective and affordable, were adopted on an ongoing basis.

### Recruitment strategies

Recruitment strategies were coordinated centrally by the study project manager (KB). In general, nationwide strategies were implemented by staff at the central coordinating centre, while local and community strategies were implemented by site study nurses and investigators. Each strategy is described in turn below. Strategy descriptions were guided by the Template for Intervention Description and Replication (TIDieR) Checklist, which lists the items to be reported when describing interventions to support replication [19].

#### Radio advertising

Radio advertising involved 30-second paid advertisements on 20 different radio stations (nine talkback stations and 11 music stations). Three different scripts were recorded over the course of the study in order to keep the message fresh (see Additional file 1). Advertisements ran from January 2014 to July 2016, but were not run continuously on all stations over this period. Instead, advertisements ran in campaigns of 3–4 weeks’ duration, with stations running between one and seven campaigns over the course of study recruitment. In total 68 campaigns were run, 45 on talkback stations and 23 on music stations, and advertisements were played in a total of 7110 paid spots. In addition to paid spots, stations offered bonus filler spots free of charge. In some cases these were more frequent than the paid spots and so the total number of times that study radio advertisements were run is likely to be in the range of 10,000–15,000 times.

In Australia, radio stations are generally broadcast within a single state so advertisements were booked separately for each of the five states where the study sites were located. Radio stations were selected based on advertising costs and listener demographics. To inform selection of advertising times, we sourced listener demographics (including age and gender) by time of day for each radio station. Generally, men aged 50 to 74 years were most likely to listen in the early mornings and late afternoons on weekdays, although these were also the most expensive times to advertise. A single campaign was booked on selected stations and the number of participants screened and enrolled, as well as the cost per participant screened and enrolled, were measured. Campaigns on stations with a cost of less than AU$50 per participant screened were generally repeated. Modifications were made to the time of day that advertising was played based on the performance of previous campaigns and on advice from radio station advertising personnel.

#### Mail-outs

Mass mail-outs involved posting a study invitation package to men on the Medicare database by the Australian Government Department of Human Services (DHS). The Medicare database, the infrastructure underpinning the national health scheme, includes Australian residents who are eligible for public healthcare, generally those who are Australian or New Zealand citizens, or have permanent Australian residency status. Mailings were conducted in July 2016 (40,000 invitations sent), September 2016 (60,000 invitations sent) and November 2016 (30,000 invitations sent), with 130,000 men in total being mailed once each. Mailing recipients were randomly selected from the Medicare database based on being male, aged 50–74 years and living within close proximity of one of the study sites (the initial mailing included a sample of men living within a 20-km radius of a study site and the subsequent mailings were further restricted to men living within 5–10 km of a study site). Men who had been prescribed testosterone or anti-diabetic therapies within the previous 12 months were excluded from the mailing list by linking to the Pharmaceutical Benefits Scheme database. The invitation package consisted of a cover letter from the DHS, an invitation letter from the study Chair and a study postcard (see Additional file 1). The mailing was conducted by a third party mailing house contracted by the DHS. The contact details of mailing recipients were kept confidential and were not shared with the study coordinating centre.

In addition, a one-time mail-out by a single network of general practices (GPs) to a targeted group of their patients was conducted from February to March 2013 in one city. Though the number of letters sent in this GP mail-out was not recorded it is estimated to be less than 500 letters.

It is possible but unlikely that men who received a letter in the GP mail-out in early 2013 also later received a letter from the DHS in 2016. The responses to the small GP mail-out and the later and much larger DHS mail-out were recorded and reported separately.

#### Television, radio and newspaper news coverage

In the period January 2013 to June 2016, approximately 15 press releases and approaches to media were made. The study chose not to engage a public relations firm due to cost concerns. Instead, press releases were facilitated by site investigators and prepared by University and Hospital media offices and were distributed to local, state and national television, radio and newspaper news organisations. Press releases highlighted newsworthy aspects of the study and provided quotes from study investigators and study participants. Media office contact details were provided so that journalists could arrange interviews with investigators and participants. Over the recruitment period, nine newspaper stories, eight television stories and seven radio news stories were broadcast.

#### Facebook promotions and advertising

The study Facebook page was set up by the central coordinating centre in May 2013. Over the period May 2013 to December 2016, 94 stories were posted to the study Facebook page. Stories covered a mixture of topics including information about the study and how to join, links to news stories about the study, men’s health information and general interest stories. In addition, 23 advertising campaigns and paid boosted posts were run intermittently in the period October 2013 to December 2016 (see Additional file 2 for definitions of common Facebook advertising terms). Advertisements focussed on inviting men to join the study. By contrast, boosted stories tended to promote news stories relating to the T4DM study. Advertisements and paid boosted stories targeted men aged 50 years or older living in a capital city with a T4DM study site. Examples of advertisements and posts can be found in Additional file 1. The Facebook Ads Manager application allowed advertising performance to be monitored in real time by reporting the number of impressions, number of clicks, cost per click and cost per 1000 impressions of each campaign. While paid campaigns were running, the coordinating centre reviewed performance statistics daily and increased or decreased the advertising spend according to the success of the advertisement. Comments from participants and from the public were used to refine Facebook page content over time.

Indirect Facebook promotions were also used. When participants completed the online screening questionnaire they were invited to share information about the study on their Facebook page. Local organisations and businesses, such as sporting clubs, social clubs and healthcare providers, were also approached to share information about the study on their own Facebook pages. We were unable to determine how many people and organisations shared information about the study on their Facebook pages.

#### Google advertising

Paid Google advertising was set up by the central coordinating centre using the Google AdWords application (see Additional file 2 for definitions of common Google advertising terms). The purpose of these advertisements was to display a link to the study website at the top of the Google search results screen when members of the public googled terms which indicated that they might be interested in joining the study. We identified four possible Google search themes: diabetes prevention, low testosterone, weight loss and nocturia (night-time urination). However, after further investigation the low-testosterone theme was rejected due to Google advertising rules and the weight-loss theme was rejected due to the high levels of competition and hence high cost. For the two remaining themes (diabetes prevention and nocturia), Google AdWords provided a list of the most commonly used related search terms, known as keywords, which we used to build our advertising campaigns (see Additional file 1). Advertising ran from July 2013 to October 2014 and October to December 2015. All advertisements were targeted to users within Australia only.

Later in the recruitment period, additional Google advertisements were run to ensure that people who searched for the T4DM study name were able to locate the website easily. Since the purpose of these advertisements was to facilitate screening of potential participants who already knew about the study rather than to promote the study to the public, these advertisements and their associated costs have not been included in this paper.

#### Newspaper advertising

In December 2013, one paid advertisement was placed in a Sunday newspaper with a circulation of 250,000 in one capital city. If effective, we planned to roll out newspaper advertising to other cities.

#### Community outreach activities

Throughout the recruitment period, site staff, and to a lesser extent, central coordinating centre staff, conducted a range of community outreach activities. These included: (1) displaying posters in local businesses, organisations, libraries and hospitals; (2) attendance at local community and health service events and (3) approaching local businesses and organisations to promote the study to their customers, members and employees. Organisations who agreed to support the study included men’s community and recreational groups, private school old boys’ associations, private health insurance companies, trade unions, government workplaces, diabetes groups and Weight Watchers ®. These organisations supported the study through a variety of means including placing information about the study in their print newsletters, email newsletters, on their websites, on Facebook pages and on notice boards. The majority of community outreach activities occurred in the first year of study recruitment (2013), but continued sporadically throughout recruitment.

In addition to these unpaid promotions, one paid promotion through a professional football club based near one study site was trialled for 1 week in June 2016. The study was featured in the club’s weekly email newsletter to its 9300 members as well as in banner and gutter ads on the club’s website.

#### Healthcare provider referrals and promotions

Throughout the recruitment period we approached local general practitioners (GPs) and pathology collection centres to support study recruitment. The central coordinating centre wrote to 1024 GPs in the areas surrounding study sites to ask them to refer suitable patients to the study and to display a study poster in their waiting room areas. Site staff also attended local GP meetings to inform them about the study. We asked pathology companies to display posters in their waiting rooms and to print information about the study on the bottom of the reports of men who might be eligible for the study based on their blood test results.

#### Recruitment strategy monitoring and enhancement

The recruitment management process involved repeated cycles of strategy implementation, monitoring and enhancement. The number of participants enrolled as a result of each strategy, the direct costs and the staff effort involved were monitored in real time and reported to the Steering Committee on a monthly basis. The Committee identified the number of participants enrolled as the primary means for assessing strategy effectiveness but also considered cost-effectiveness, staff effort, potential to reach large numbers of men or to be targeted to men who were most likely to be eligible.

### Outcomes and data analysis

#### Strategy attributes

Attributes which were thought to impact recruitment results were described for each strategy: content format (text, image, audio, audio-visual), content length (short, medium, long), approach to prospective participants (direct, indirect), level of targeting (ability to reach members of the public who were most likely to be eligible for the study in terms of age, location and health), potential reach (the number of people who would see the strategy), frequency of exposure, and whether or not the strategy included an online component.

#### Strategy exposure and contribution

Where possible, we recorded the number of people exposed to each strategy. In addition, all men completing the pre-screening questionnaire were asked to report how they heard about the study. This information was linked to the participant’s screening and enrolment status by a unique participant identifier to estimate the contribution of the strategy (the percentage of all screened and randomised participants contributed by each strategy) and to estimate how many participants heard about the study through online and traditional sources.

#### Strategy cost

The direct cost of implementing each recruitment strategy was recorded. The direct cost did not include staffing costs or the cost of conducting screening and enrolment activities. Costs were recorded in Australian dollars and were adjusted for inflation using the Australian Consumer Price Index [33]. All costs in this paper are expressed in June 2018 terms.

We determined that measuring the indirect cost of each strategy would not be feasible. Instead, we collected detailed feedback from recruitment staff (at the central coordinating centre and at study sites) in order to estimate the staff effort involved in implementing each strategy (categorised as low, moderate or high per participant enrolled).

#### Overall strategy appraisal

For each strategy, the number of participants randomised, the direct cost per participant, and the staff effort per participant were estimated and each scored 0 (lowest) to 3 (highest). Since the number of participants randomised was identified as the most important outcome, this was the primary means of assessing the effectiveness of each strategy (highly effective, effective, moderately effective, limited effectiveness, ineffective). However, the direct cost per enrolment, level of staff effort required per enrolment, and the strategy’s attributes were also considered to come to a final subjective appraisal of each strategy.

#### Statistical methods

Data analysis was conducted in SAS v 9.4 (Cary, NC, USA). Simple descriptive statistics were used to describe the number of participants screened and randomised. Differences between groups in enrolment rates and in the proportion aged 60 years or older were tested using chi-square analyses with a significance level of 5%.

## Results

### Overall study recruitment

**Fig. 1:**
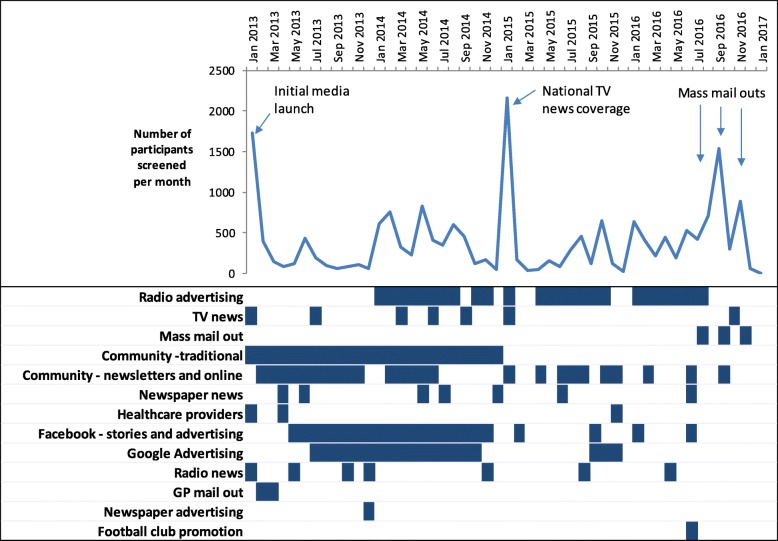
Number of participants screened, and recruitment strategies conducted, by calendar month

During the recruitment period (January 2013 to February 2017) 19,022 men were screened and 1007 were randomised to the T4DM trial. The number of men screened per month fluctuated over the recruitment period (Fig. 1), most likely influenced by the mix of promotional activities occurring at the time. Spikes in the number of men screened were observed when the trial was featured in media news stories and when radio advertising campaigns and mass mail-outs were being conducted.

### Evaluation of promotional activities

#### Number of participants recruited

Table 2 shows the number of men screened and randomised as a result of each promotional activity. Almost 80% of participants heard of the study through one of three methods; radio advertising (42% of participants), television news coverage (20%) or mass mail-outs (17%). No other single strategy contributed more than 4% of all enrolments. Excluding strategies that resulted in fewer than ten randomisations, the randomisation rate (percentage of screened participants who went on to be randomised) did not differ between strategies (*p* = 0.31). The average randomisation rate was 5%.

**Table 2:** How screened and randomised participants reported hearing about the study

How men reported hearing about the study^1^	Description of associated recruitment promotions^2^	# Screened	# Randomised (%)^3^	Contribution (%)^4^
Radio advertising	7110 × 30-s paid advertisement placements across 20 radio stations	7667	418 (5%)	42%
TV news	8 television news stories (6 national and 2 in single cities)	4127	202 (5%)	20%
Mail-out by DHS	130,000 invitation letters posted to government mailing list	3211	173 (5%)	17%
Community promotions	Posters, community events, promotion on other organisations’ websites, newsletters and Facebook pages	998	43 (4%)	4%
Word of mouth (not otherwise specified)	N/A	491	34 (7%)	3%
Newspaper news	9 newspaper stories (3 major newspapers, 3 local newspapers, 2 online news sites, 1 professional magazine)	622	31 (5%)	3%
Healthcare provider	1024 GP clinics mailed, attendance at GP events, distribution of posters to pathology collection centres, GPs, clinics and hospitals	450	29 (6%)	3%
Facebook	94 unpaid Facebook posts, 23 paid Facebook advertisements and boosted posts, requests to participants and organisations to share study on Facebook	369	16 (4%)	2%
Other internet	Three Google AdWords campaigns, study website, links on other websites	410	15 (4%)	1%
Radio news	7 radio news stories (all in single cities)	182	10 (5%)	1%
Mail-out by GP	Invitations mailed from GP clinic near to one study site. Number of invitations sent not known	47	1 (2%)	0%
Newspaper advertising	1 advertisement in a Sunday paper in 1 city	33	1 (3%)	0%
Football club promotion	Email newsletter and 1 week of website advertising at one football club near to 1 study site	5	0 (0%)	0%
Not specified	N/A	410	34 (8%)	3%
Total		19,022	1007 (5%)	100%

^*DHS* Department of Human Services, *GP* general practitioner, *N/A* not applicable^

^1^Where a participant reported hearing about the study from multiple sources only the primary source is shown

^2^Unless otherwise specified, strategies were implemented across all study sites

^3^Percentage of screened participants who went on to be randomised to the study

^4^Contribution defined as the percentage of all participants randomised who were randomised from a particular source

The response rate to the mass mail-out by the DHS was 2.5% (3211 men screened from 130,000 letters sent); 173 of these men went on to be randomised (0.1% of all men mailed). It was not possible to calculate the response rate for other recruitment strategies; for example, radio advertising and news stories, since the denominator number of men exposed to these strategies was unknown.

#### Recruitment cost

A total of AU$598,633 was spent on promotional activities at an average cost of AU$31 per participant screened and AU$594 per participant randomised. The total direct cost and cost per participant for each strategy are shown in Table 3. The cost of individual strategies ranged from no cost (free media news coverage and word of mouth) to AU$312 per screened participant for online promotion of the study by a football club. Of the paid strategies, mass mail-out by the DHS was the most cost-effective (AU$40 per screening and AU$745 per randomisation).

**Table 3:** Direct cost of recruitment strategies

Recruitment strategy^2^	Total direct cost^1^	Cost per screening	Cost per randomisation
Radio advertising	$451,705	$59	$1081
Mail-out by DHS	$128,968	$40	$745
Community promotions	$122^3^	N/A^4^	N/A^4^
Healthcare provider	$1272^3^	N/A^4^	N/A^4^
Facebook	$10,029	N/A^4^	N/A^4^
Google advertising	$1931	N/A^4^	N/A^4^
Mail-out by GP	$1104	$23	$1104
Newspaper advertising	$1941	$59	$1941
Football club promotion	$1561	$312	N/A^5^
Total	$598,633	$31	$594

*DHS* Department of Human Services, *GP* general practitioner, *N/A* not applicable

^1^All costs are expressed in Australian dollars. Costs have been adjusted for inflation and are expressed in June 2018 prices

^2^Excluding strategies that did not involve any direct cost (TV, radio and newspaper news coverage, word of mouth)

^3^Cost of printing and posting posters. Community promotions and contact with healthcare providers was predominantly free of direct cost

^4^Where it was not possible to differentiate participants enrolled through paid and unpaid activities; for example, paid Facebook advertising vs unpaid sharing of Facebook posts, a cost per screening and randomisation is not reported

^5^No participants were randomised as a result of this strategy. The cost per randomisation could not be calculated

#### Staff time and effort

The task of organising, conducting and monitoring promotional activities took an estimated average of 20 person-hours per week over the 4-year recruitment period. The work was divided between seven study team members (one project manager at the central coordinating centre and six site-based study nurses) and fluctuated throughout the recruitment period. The staff effort required for each strategy, proportional to the number of participants enrolled, is estimated in Table 4. In general, paid strategies, such as advertising and mass mail-outs, required the least staff effort, while low-cost and unpaid strategies, such as community activities, required the most staff effort.

**Table 4:** Promotional strategy atttributes, outcomes and appraisal of effectiveness

	Attributes						Assessment of outcomes			
Promotion	Format^1^, length^2^	Direct^3^	Targeted^3^	High reach^3^	Frequent^3^	Online component	Contributed to enrolment^3^	Direct cost per participant^4^	Staff effort per participant^4^	Appraisal of effectiveness^5^
TV news and current affair coverage	Audio-visual, medium			+++	+	Yes	+++		– –	Highly effective Advantages: no cost, very high reach, audio-visual format, credible source Disadvantages: single exposure, challenging to arrange
Mass mail-out by DHS	Text + image, long	+++	++	+++	+	No	+++	– –	–	Highly effective Advantages: direct, targeted, high reach, credible source Disadvantages: administrative process for approval, cost
Radio advertising	Audio, short		+	+++	+++	No	+++	– – –	–	Highly effective Advantages: high reach and frequency Disadvantages: costly, short audio-only format
Newsletter mentions: businesses and community organisations	Text (+ image), usually short	+	+	++	+	Yes	++		– –	Effective Advantages: no cost, moderate reach, credible source, potential to identify influencer/champion within organisation Disadvantages: challenge of identifying willing organisations
Word of mouth	UNK	+	UNK	+	UNK	UNK	++			Effective Advantages: no cost, trusted source Disadvantages: usually incidental
Newspaper articles: print and online	Text (+ image), medium			+++	+	Yes	++		– –	Effective Advantages: high reach (although shrinking), offline and online options, credible source Disadvantages: single exposure, challenging to arrange
Publicly displayed posters	Text + image, medium		+	+	++	No	++		– – –	Effective Advantages: low/no cost, simple, local to centres Disadvantages: small potential reach, time-consuming
Online promotion: businesses and community organisations	Text (+ image), short		+	++	+	Yes	+		– –	Moderately effective Advantages: no cost, can be a credible source Disadvantages: single exposure, challenging to arrange
Radio news coverage/interviews	Audio, medium			+++	+	No	+		– –	Moderately effective Advantages: high reach, credible source Disadvantages: audio-only, single exposure, challenging to arrange
Referral by GP	Face-to-face	+++	+++	+	+	No	+		– –	Limited effectiveness Advantages: direct and very targeted, a trusted medical source Disadvantages: limited reach, challenging to seek referrals from health professionals not affiliated with trial
Direct approach/invitation from study centre	Text, long	++	+++	+	+	No	+		– – –	Limited effectiveness Advantages: direct and very targeted, a trusted medical source Disadvantages: very limited reach, challenging to identify potential participants through hospitals due to nature of trial
Referral by pathology service (printed on bottom of path results)	Text, short	++	+++	+	+	No	+		– –	Limited effectiveness Advantages: direct and very targeted, a trusted medical source Disadvantages: limited reach, challenging to arrange
Organic Google search	Text, short		++	++	UNK	Yes	+		–	Limited effectiveness Advantages: no cost Disadvantages: limited reach due to nature of trial
Paid Google search	Text, short		+++	+	UNK	Yes	+	–	– –	Limited effectiveness Advantages: potentially high reach, affordable compared to other paid strategies, flexible Disadvantages: technically challenging, potential limited by the nature of trial
Facebook-paid ad	Text + image (+ audio-visual), short		++	++	+++	Yes	+	–	– –	Limited effectiveness Advantages: affordable compared to other paid strategies, potential to use images and video, high frequency, flexible Disadvantages: technically challenging, limited engagement with trial demographic
Community events: presentation/stand	Mixed	++	++	+	+	No	–		– – –	Ineffective Advantages: direct and potentially targeted Disadvantages: very limited reach. Time-consuming
Newspaper advertisement: print	Text + image, short			++	++	No^6^	–	– – –	–	Ineffective Advantages: Potential to use images Disadvantages: Costly, falling reach
Mass mail-out by GP	Text, long	+++	+++	+	+	No	–	– – –	–	Ineffective Advantages: direct and very targeted, a trusted medical source Disadvantages: limited reach, costly
Unpaid post on study Facebook page	Text + image, short	+	+	+	++	Yes	–		– – –	Ineffective Advantages: no cost Disadvantages: limited engagement with trial demographic, time-consuming if done with high frequency

^1^Format categorised as text, image, audio, audio-visual, face-to-face or mixed

^2^Length categorised as short, medium or long

^3^+++ = to a great extent, ++ = somewhat, + = a little, [blank] = not at all, UNK = unknown

^4^ – – – = high, – – = moderate, – = low, [blank] = none

^5^Qualitative judgement of the effectiveness (in terms of the estimated number of participants enrolled), advantages and disadvantages of each strategy

^6^Online newspaper advertising is a possible recruitment strategy but was not used in this study

#### Overall strategy appraisal

Table 4 describes each strategy’s attributes, its ratings for the three key outcomes (number of participants enrolled, direct cost and staff effort), and a subjective appraisal of the advantages and disadvantages of the strategy. While radio advertising, television news coverage and mass mail-outs were identified as the most effective recruitment strategies, each of these strategies had at least one disadvantage in terms of cost, frequency or format.

#### Response to online recruitment strategies

Eight hundred and thirty-one people liked the study Facebook page, but engagement with content posted on the Facebook page was generally low. Unpaid posts usually received less than five likes (mostly from study staff) and few, if any, comments. Facebook-paid advertisements and boosted posts cost AU$10,029 and received 2,473,966 impressions, resulting in 21,477 clicks or other engagements. The average cost per click was AU$0.47 and the average cost per 1000 impressions was AU$4.05.

The results of the Google advertising campaigns are shown in Table 5. AU$1931 was spent on Google advertising to promote study participation, resulting in advertisements being displayed 57,202 times and clicked on 5939 times. The average click-through rate was 10% and the average cost per click was AU$0.33. We did not record how many of the people who clicked on an advertisement went on to be screened and randomised to the T4DM study.

**Table 5:** Results of Google advertising campaigns

Campaign	Date range	Maximum cost per click bid^1^	# Clicks^2^	# Impressions^3^	Click-through rate^4^	Total cost^5^	Average cost per click
Diabetes prevention: Campaign 1	Jul 13—Oct 14	Auto^6^: $1.01	4940	46,325	10.66%	$1040	$0.21
Diabetes prevention: Campaign 2	Oct 15	$2.00	684	4971	13.76%	$356	$0.52
Nocturia campaign	Oct 15—Dec 15	$3.00	315	5906	5.33%	$535	$1.70
Total			5939	57,202	10%	$1931	$0.33

^1^An amount set by the advertiser as the maximum amount they are willing to pay per click. The actual amount paid may be less than this depending on how much other advertisers have bid

^2^Number of times a user clicked on the link within an advertisement

^3^The number of times that an advertisement was shown on screen

^4^The number of times an ad was clicked on, divided by the total number of times the ad was shown

^5^All costs are expressed in Australian dollars. Costs have been adjusted for inflation and are expressed in June 2018 prices

^6^The maximum bid for this campaign was set automatically by Google AdWords to optimise results

In total, 1433 participants (8%) of participants reported hearing about the study online. However, this is likely to be an underestimation since some sources had online and offline components; for example, organisations promoted the study by publicly displaying posters and posting information on their websites, and it was, therefore, not always possible to determine whether a participant’s information source was online or offline. In younger participants (aged < 60 years) 7% reported using an online source compared to 8% in older participants (aged ≥ 60 years). The proportion of participants hearing about the study online did not differ by age (*p* < 0.28).

## Discussion

The three most effective recruitment strategies were: (1) repeated bursts of high-frequency, targeted radio advertising, (2) infrequent but high-reach television news reports (3) direct, mass-mailed invitations from a credible government health agency. Other promotional strategies, including newspaper and radio news coverage, newspaper advertising, publicly displayed posters, attendance at local community events, mentions in email and posted newsletters, and promoting the study online through Facebook, Google and other websites, collectively accounted for less than 20% of all randomisations. These findings are broadly consistent with those reported by other contemporary RCTs recruiting men aged over 50 years [16, 17, 34]. While previous studies reported that community outreach activities, such as displaying posters in the local community and attending community events, were ineffective [16, 17, 34], we achieved moderate success by expanding our community outreach activities to encompass online promotion through organisations’ email newsletters, websites and Facebook pages. While the numbers of resulting participants were small, the fact that these online approaches involved no direct cost and little staff effort made them a worthwhile component of the overall recruitment strategy mix. Unlike previous studies [16, 35, 36], we found that newspaper advertising was not an effective strategy and so this strategy was abandoned after the placement of only a single advertisement. This may be explained by the fall in print newspaper readership over recent years [37]. However, this finding should be interpreted with caution as it was based on the response to a single newspaper advertisement in a single city. Overall, the average promotion cost per randomised participant was AU$594 with mass mailing the most cost-effective of the paid promotional strategies. This cost compared favourably with promotional costs in a large-scale RCT in diabetes prevention [9], and an RCT in testosterone supplementation in older men [34], which are estimated to have cost at least AU$2700 per participant in June 2018 terms. It should be noted that these RCTs had different target populations and eligibility criteria which may account in some part for their higher recruitment promotion costs.

Our efforts to recruit participants using Facebook and Google advertising achieved disappointing results. Like others [24, 28, 38], we found such online advertising fast and flexible to implement, and easy to monitor in real time. However, unlike studies recruiting predominantly younger people [21, 25] and women [27, 28], our Facebook advertising campaigns resulted in few enrolments. While large numbers of men aged over 50 years use Facebook [39], their engagement with the study content on Facebook was low. This was likely to have constrained the reach and impact of the study’s Facebook promotions. It was unclear whether this lack of engagement was due to a deficiency in the content we posted or due to men in this age group’s social media habits more generally [38]. Future research could address this uncertainty by using the randomised split-testing capabilities built into the Facebook advertising interface to evaluate men’s responses to variations in content messaging and images [25, 28]. We also observed a disappointing response to our Google advertising campaign. We hypothesise that this failure was due to the nature of the study question focussing on diabetes prevention. Prospective participants may not have been aware that they were at an increased risk of developing diabetes. We presume they were, therefore, unlikely to search in Google for terms relating to diabetes prevention and pre-diabetes, limiting the reach of our Google advertisement. By contrast, studies that were able to define study-specific search terms, for example, relating to cessation of smokeless tobacco [22] or depression [23], reported that Google advertising was an effective and affordable recruitment strategy.

### Limitations and areas for future research

Despite being rigorously conducted, the analyses presented in this paper are based on observational data. The results may be confounded by differences in the timing and target location of recruitment strategies, which we were not able control for in the analyses. Furthermore, the individual promotional strategies, by their nature, involved differences in the form, length and style of content. It is possible that differences in the observed responses to strategies were due, in some part, to these content differences rather than the promotional strategies themselves. The results observed in this study may not be generalisable to other RCTs due to the possible impact of differences in disease area, target population, study design and location.

Another limitation of this study was the difficulty in accurately measuring the contribution of each strategy to enrolment. This difficulty was two-fold. Firstly, some participants supplied insufficient information to pinpoint a specific recruitment strategy. For example, if the source of information was reported as ‘GP’ then it was unclear whether the participant was referred to the study by their GP, or whether they saw a study recruitment poster in the GP’s waiting room. Since we knew where and when particular strategies were being conducted, we cross-referenced the participant’s location and date of screening to resolve these uncertainties wherever possible. Secondly, for practical reasons we coded only a single recruitment source for each participant. However, it was evident from the optional, free-text responses provided by some participants, as well as from speaking directly to study participants, that some participants heard about the study through multiple sources. In such cases, the marginal contribution of these multiple strategies could not be measured, possibly influencing the estimates of effectiveness. To address these challenges, future studies could adapt digital marketing techniques, such as custom Universal Resource Locator (URL) tracking and Google Analytics goals, to more accurately track the sources of participant recruitment. Future researchers could also conduct participant interviews early in the recruitment phase to understand the media habits and preferences of the target population and the possible impact of multiple promotional sources. Such marketing activities may require specialised skills and additional resources. Trial recruitment managers face the challenge of straddling the divide between the methodological rigour of clinical trial research and the ‘move fast and break things’ culture of digital marketing [40]. The best approach to combining these divergent paradigms is still to be determined, particularly given the ethical standards and oversight required in RCT recruitment [38].

A trial-and-error approach to optimising recruitment promotions is likely to increase recruitment costs and result in recruitment delays, yet, in the past, trial recruitment managers had little other choice due to the lack of reliable evidence. This observational evaluation presents an approach for selecting, implementing, monitoring and enhancing recruitment promotional activities. We hope that future trials can adapt and improve on this approach to meet their recruitment targets.

## Conclusion

The most effective strategies to recruit men aged 50–74 years to the T4DM diabetes prevention RCT were repeated bursts of high-frequency radio advertisements supported by occasional television news coverage and mass mail-outs by a government health agency. Close monitoring of recruitment outcomes and ongoing enhancement of recruitment activities played an important role in overcoming the recruitment challenges in this RCT. This paper provides future researchers with estimates of the effectiveness of a range of traditional and online promotional strategies as well as presenting an approach to collecting and analysing promotional strategy recruitment metrics.

## Additional files

Additional file 1: Examples of various promotional materials used throughout the T4DM diabetes prevention study. (PDF 2004 kb)

Additional file 2: Definitions of common Facebook and Google terms. (PDF 325 kb)

## Data Availability

The datasets used and analysed during the current study are available from the corresponding author on reasonable request.

## Abbreviations

DHS: Department of Human Services
GP: General practitioner/general practice
RCT: Randomised controlled trial
T4DM: Testosterone for the Prevention of Type 2 Diabetes study
TIDieR: Template for Intervention Description and Replication Checklist
URL: Universal Resource Locator
