# Supplementary material for: Recruitment of men to a multi-centre diabetes prevention trial: an evaluation of traditional and online promotional strategies
Source: Trials. 2019 Jun 19;20:366. doi: 10.1186/s13063-019-3485-2 (PMC6585027; doi:10.1186/s13063-019-3485-2)
Supplement: Supplementary file 1 — Examples of various promotional materials used throughout the T4DM diabetes prevention study. (PDF 2004 kb) [file 13063_2019_3485_MOESM1_ESM.pdf]

# Additional file 1: Examples of various promotional materials used throughout the T4DM Diabetes Prevention Study

## Radio advertising

### *T4DM Study Radio Advertisement Script #1*

So you’ve hit your fifties and your six pack is now a keg, you’re not sleeping well and you need a whizz in the night. And you can’t find your mojo when you need it most. Well you might have low testosterone and be at risk of developing Type 2 Diabetes. Now is the chance to join a research trial with a proven weight loss program that is testing the effect of testosterone treatment to prevent progression to type 2 diabetes. So if you’re aged between 50 to 74 you might be a suitable candidate for the study. Find out more at diabetesprevention.org.au. You’ve nothing to lose except excess weight and you might even find your mojo.

### *T4DM Study Radio Advertisement Script #2*

[Sound of ticking clock] That’s time ticking away. You’re over fifty. Are you really going to spend the rest of your life leaning over that gut just to see if your shoes are shiny? You could have low testosterone and be at risk of developing Type 2 Diabetes. It’s time to get your strut back! Join a research program which may prevent progression to diabetes. A free Weight Watchers program is also included. If you’re male, aged 50 to 74 see diabetesprevention.org.au.

### *T4DM Study Radio Advertisement Script #3*

New Year, new man! Right? Wrong! You’re over fifty now, you’re tired, maybe you’ve lost your mojo. You might be at risk of Type 2 Diabetes and have low testosterone. Now is the chance to join a research trial with a proven weight loss program that is testing the effect of testosterone treatment to prevent progression to diabetes. If you’re aged between 50 and 74 why not make it a resolution to join the study today? At diabetesprevention.org.au. New Year, new man? You betcha!

## Google Adwords ads and associated keywords

### *Diabetes prevention advertisement*

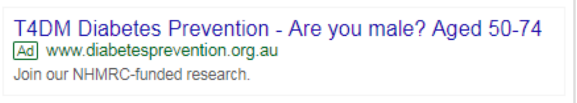


Keywords:

diabetes prevention, diabetes type 2 prevention, how to prevent diabetes, preventing diabetes, pre-diabetes, prevent diabetes, prevention of diabetes type 2, to prevent diabetes, how to prevent from diabetes, prevention for diabetes, diabetes prevent, prevention of diabetes, diet to prevent diabetes, prevention of type 2 diabetes, pre-diabetic, preventing type 2 diabetes, testosterone diabetes, prevent diabetes type 2, preventing diabetes type 2, how prevent diabetes, how to prevent diabetes type 2, type 2 diabetes prevention, prevent type 2 diabetes, ways to prevent diabetes, diabetes how to prevent, prevent of diabetes, prevent diabetes diet, how do you prevent diabetes, how to prevent type 2 diabetes, catalyst, prediabetes, how do i prevent diabetes, type 2 diabetes information, how to prevent diabetes 2, how do you prevent type 2 diabetes, causes of diabetes, prediabetic, SEN diabetes

### *Nocturia advertisement*

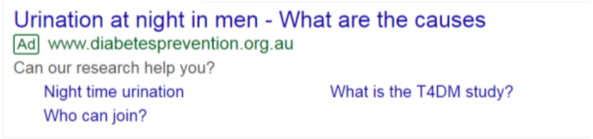


Keywords:

urinating at night, frequency urination at night, frequency of urine at night, excessive urination at night, urine frequency at night, night time urination, constant urination at night, urine at night, urination frequency at night, why do i get up at night to urinate, frequency of urination at night, urge to urinate at night, urinating at night while sleeping, normal urination frequency at night, excessive night urination, more urine at night, night urination frequency, excessive urine at night, excessive urination at night is called, urinate at night, night time urination frequency, over urination at night, frequency in urination at night, urinate more at night

## Facebook advertisements and boosted posts

**T4DM Diabetes Prevention Study**
Sponsored ·

A research study for men aged 50-74 looking at testosterone, weight and pre-diabetes

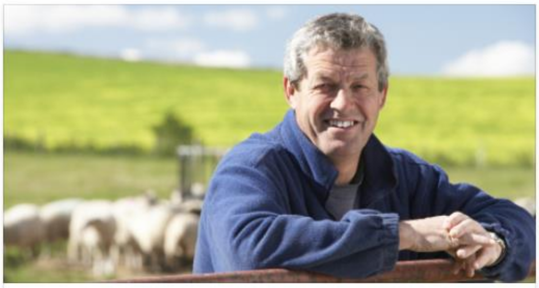

Government-funded study
Listen to our new ad to see if T4DM could be right for you.
WWW.DIABETESPREVENTION.ORG.AU

**T4DM Diabetes Prevention Study**
Sponsored ·

If you are male, overweight and aged 50-74, our research study could be for you.

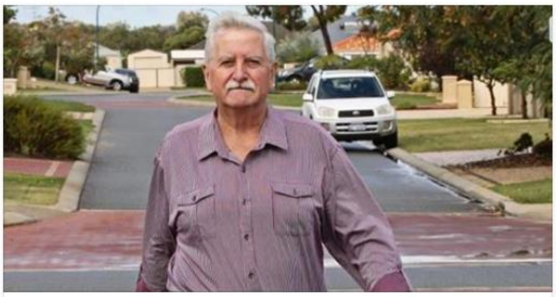

Focus on men's health
We're investigating whether a testosterone boost could reduce the risk of diabetes and other health problems. Join today!
WWW.DIABETESPREVENTION.ORG.AU
Learn More

**T4DM Diabetes Prevention Study**
Sponsored ·

We're looking for Perth men aged 50-74 to join our diabetes prevention study

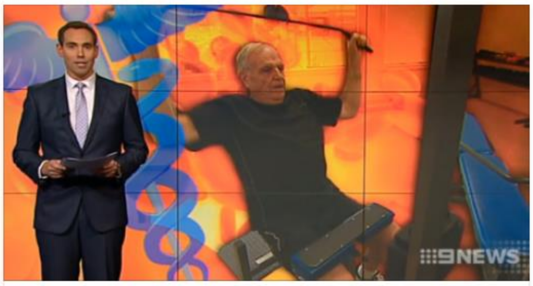


Channel 9 News
We're running this study in Perth also, watch the interview to find out more.
WWW.DIABETESPREVENTION.ORG.AU
Learn More

**T4DM Diabetes Prevention Study**
Sponsored ·

Poor sleep, obesity and low testosterone - what's the story?

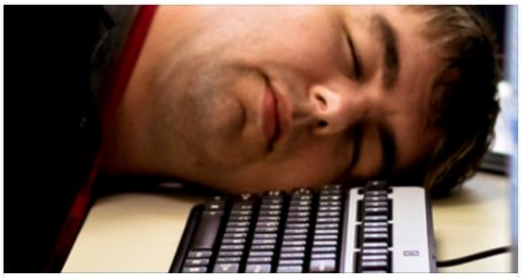

Men's health research
Our study chair looks at the links and explains how our government-funded research could help you.
DIABETESPREVENTION.ORG.AU
Learn More

**T4DM Diabetes Prevention Study**
Sponsored · Edited ·

T4DM on the news in South Australia this week. What a great boost for study. And thanks to our participant Graham for appearing in the intervi
https://www.youtube.com/watch?v=-4uX_dUfwhw&feature=youtu.be

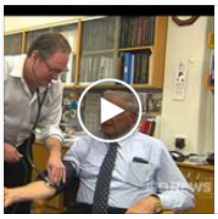

Diabetes Study | 9 News Adelaide
Researchers at the University of Adelaide are exploring whether a simple injection could stop diabetes in its tracks.
YOUTUBE.COM

**T4DM Diabetes Prevention Study**
Sponsored ·

"It's not inevitable that sex drive and erectile function wane with increasing age. And getting up at night more than once to pass urine doesn't have to be the norm either."
Our study chair, Prof Gary Wittert, is writing about men's health issues today. Read more here: http://goo.gl/sJebIQ

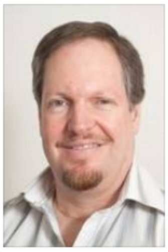

**T4DM Diabetes Prevention Study**
Sponsored ·

Like Page

Testosterone, obesity & risk of diabetes: what's the link? How could our research help?

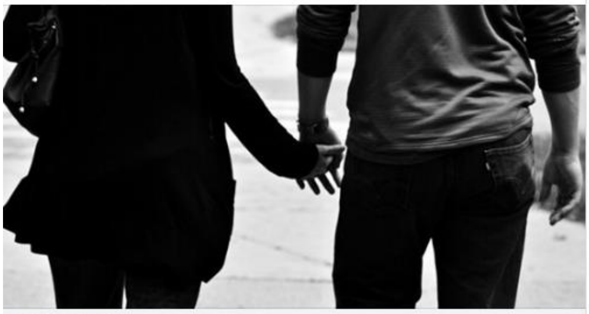

WWW.2GB.COM

**Talking to Steve Price**

Listen to an interview with our study chair, Prof Gary Witt...

Learn More

**T4DM Diabetes Prevention Study**
Sponsored ·

Like Page

Join our government-funded research today and put your health first.

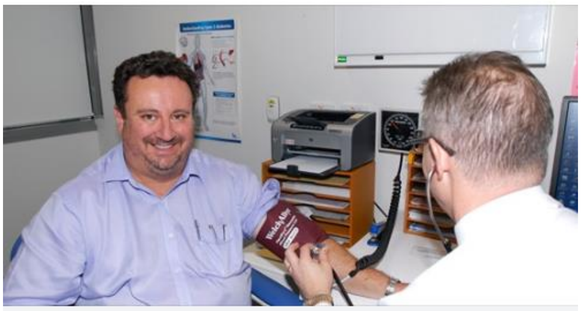

WWW.9NEWS.COM.AU

**Mens health research**

Men aged 50-74: join our research into testosterone, wei...

Learn More

**T4DM Diabetes Prevention Study**
Sponsored ·

"Being overweight can directly affect erectile dysfunction by lowering testosterone levels." An interesting article from the Sydney Morning Herald.

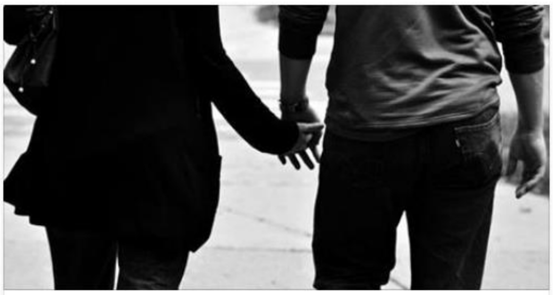

Lose weight for better sex

With obesity rates on the rise, the effects of being overweight have attracted increasing attention, but one aspect of this problem is too often overlooked - the impact on male sexual dysfunction.

SMH.COM.AU

**T4DM Diabetes Prevention Study**
Sponsored ·

Only 4 weeks left to get your screening blood tests done. We have 913 men enrolled on the study already and we'd love you to help us reach 1000. Visit www.t4dm.org.au for more details.

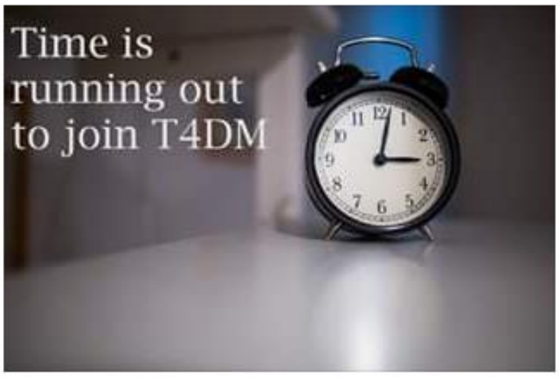

## TV news coverage

Please note that the links listed below are to third party websites not maintained by the authors. These links may not remain permanently available.

Channel 9 news Adelaide, June 2014

ABC Catalyst, September 2014

Channel 7 news, Perth, July 2013

## Posters

### *1st poster*

(poster was modified for each site to include local contact details and tear off strips)

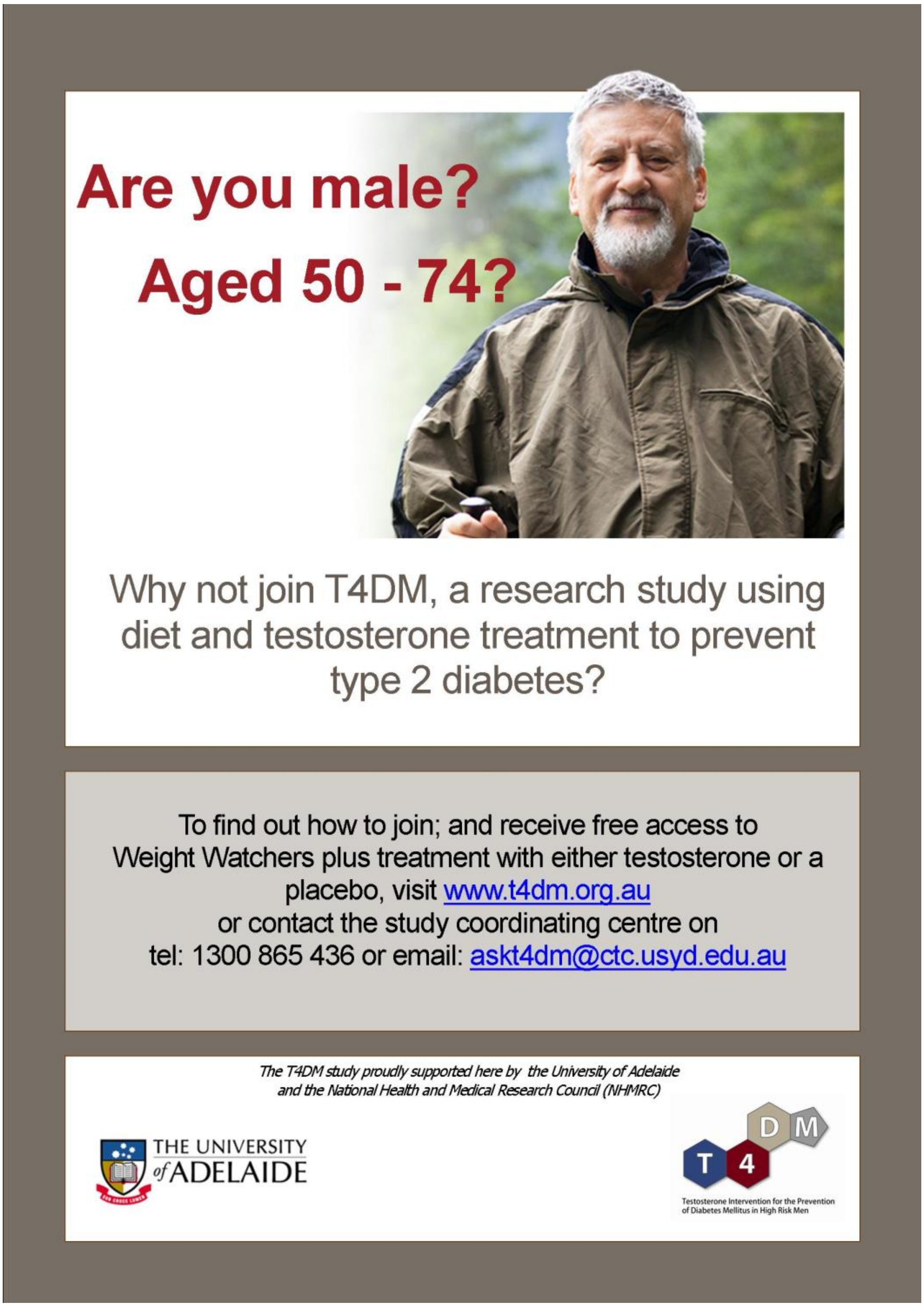

*2nd poster*

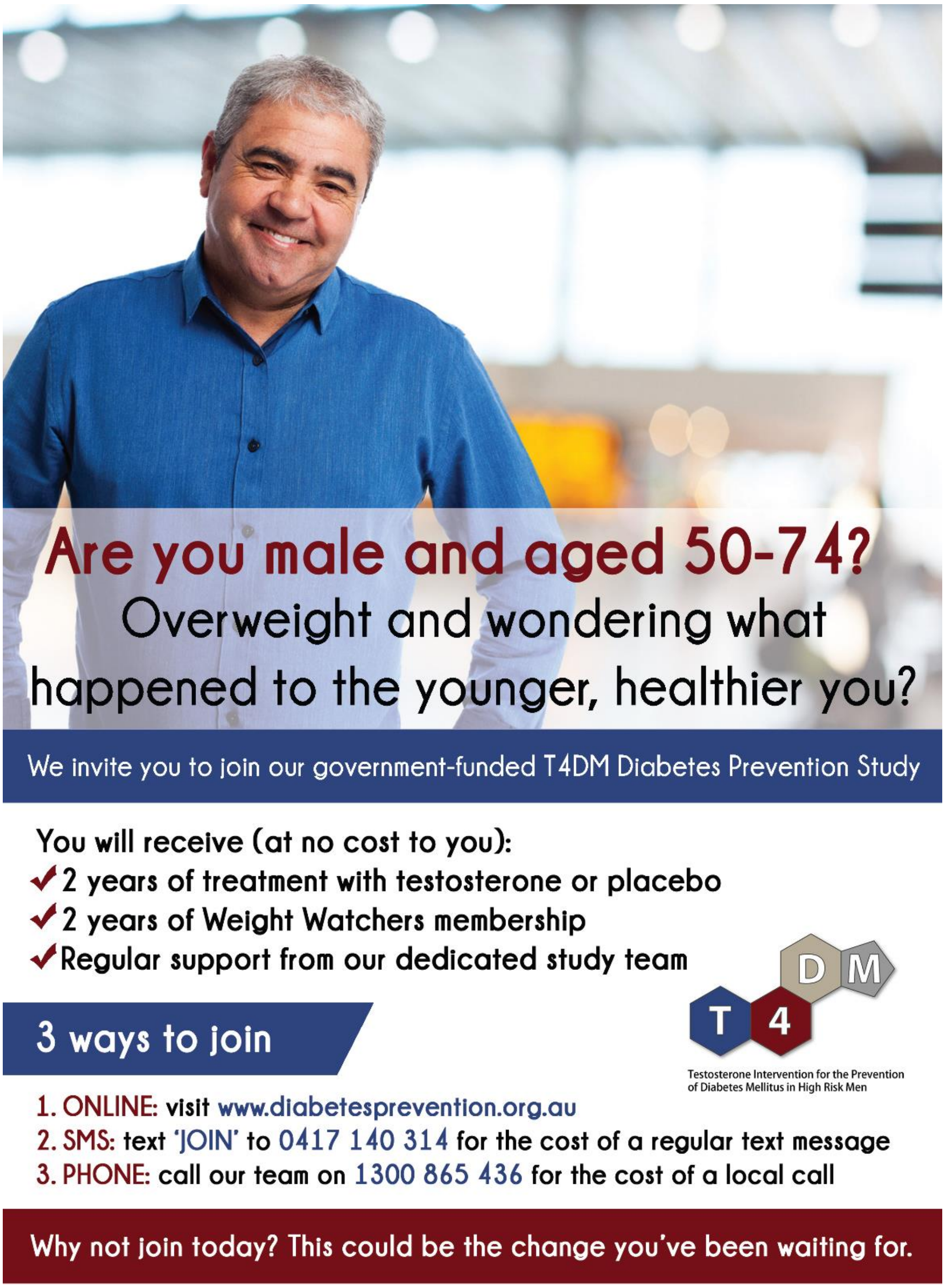
Are you male and aged 50-74?
Overweight and wondering what happened to the younger, healthier you?
We invite you to join our government-funded T4DM Diabetes Prevention Study
You will receive (at no cost to you):
2 years of treatment with testosterone or placebo
2 years of Weight Watchers membership
Regular support from our dedicated study team
T 4 D M
Testosterone Intervention for the Prevention of Diabetes Mellitus in High Risk Men
3 ways to join
1. ONLINE: visit www.diabetesprevention.org.au
2. SMS: text 'JOIN' to 0417 140 314 for the cost of a regular text message
3. PHONE: call our team on 1300 865 436 for the cost of a local call
Why not join today? This could be the change you've been waiting for.

## Mail out by Department of Human Services

(N.B. the mail out also included a cover letter from the Department of Human Services which provided information on privacy and ethical approval)

[Date]

Dear Sir

## The T4DM Diabetes Prevention and Testosterone Study

You are invited to participate in a research study which is investigating the use of testosterone to reduce the risk of diabetes in men who are at high risk of developing diabetes.

### What is the T4DM study?

The T4DM study aims to recruit 1500 men around Australia. All men who join the study will receive a 2 year Weight Watchers membership at no cost to them. Half will receive 3-monthly injections of testosterone and the other half will receive injections of placebo. Participants will not be told which treatment they are receiving.
Participants attend 11 clinic visits over a 27 month period. For more information on what is involved please visit our website (www.diabetesprevention.org.au).

### Who can join the T4DM study?

You can join the study if you are a man aged 50-74 years who is overweight or obese (if you have a waist circumference of 95cm or more).

We are looking for men who have impaired glucose tolerance and lower than normal testosterone. However, we'll check these as part of our screening process so don't worry if you don't know your glucose and testosterone levels.

Unfortunately you will not be able to join if you:

- already have type 2 diabetes,
- have a history of any cancer (except skin cancer),
- have another serious medical condition that might make the study unsafe for you (complete our online questionnaire or contact us for more info).

### Who is doing this research?

The T4DM study is run by the University of Adelaide in collaboration with the National Health and Medical Research Council Clinical Trials Centre and is conducted at 6 hospitals around Australia by senior endocrinologists. The research is majority funded by the National Health and Medical Research Council.

### How to join

You can sign up today by filling in our online screening questionnaire at:

www.diabetesprevention.org.au

Click on the Join Now button.
To find out more, contact us on askt4dm@ctc.usyd.edu.au or 1300 865 436.

T4DM STUDY COORDINATING CENTRE:
NHMRC Clinical Trials Centre
Locked Bag 77
CAMPERDOWN NSW 1450
ABN 34 093 854 267

The University of Sydney

Telephone:1300 865 436
Email: askt4dm@ctc.usyd.edu.au
www.diabetesprevention.org.au

ACN 093 854 267

**What if you have previously signed up for the study?**

The study has been running for 3 years already. You may have heard about us on the radio in the past. If you have previously completed our online screening questionnaire but haven't yet been for lab screening it isn't too late to join up. Let us know at askt4dm@ctc.usyd.edu if you need another copy of your lab screening forms.

Thank you for considering this invitation to participate in this important men's health research. Our aim at the T4DM study is to improve the health of Australian men while also supporting our study participants to lose weight and improve their health.

If you do decide to join the study we will provide you with a participant information sheet containing further details about the study. If you are not interested in being part of the study you are under no obligation to complete our online screening questionnaire.

Kind regards

Prof Gary Wittert MBBch, MD, FRACP, FRCP
Head, Discipline of Medicine
Director, Freemasons Foundation Centre for Men's Health Research
University of Adelaide

T4DM study coordinating centre
Email: askt4dm@ctc.usyd.edu.au
Tel: 1300 865 436

Postcard front

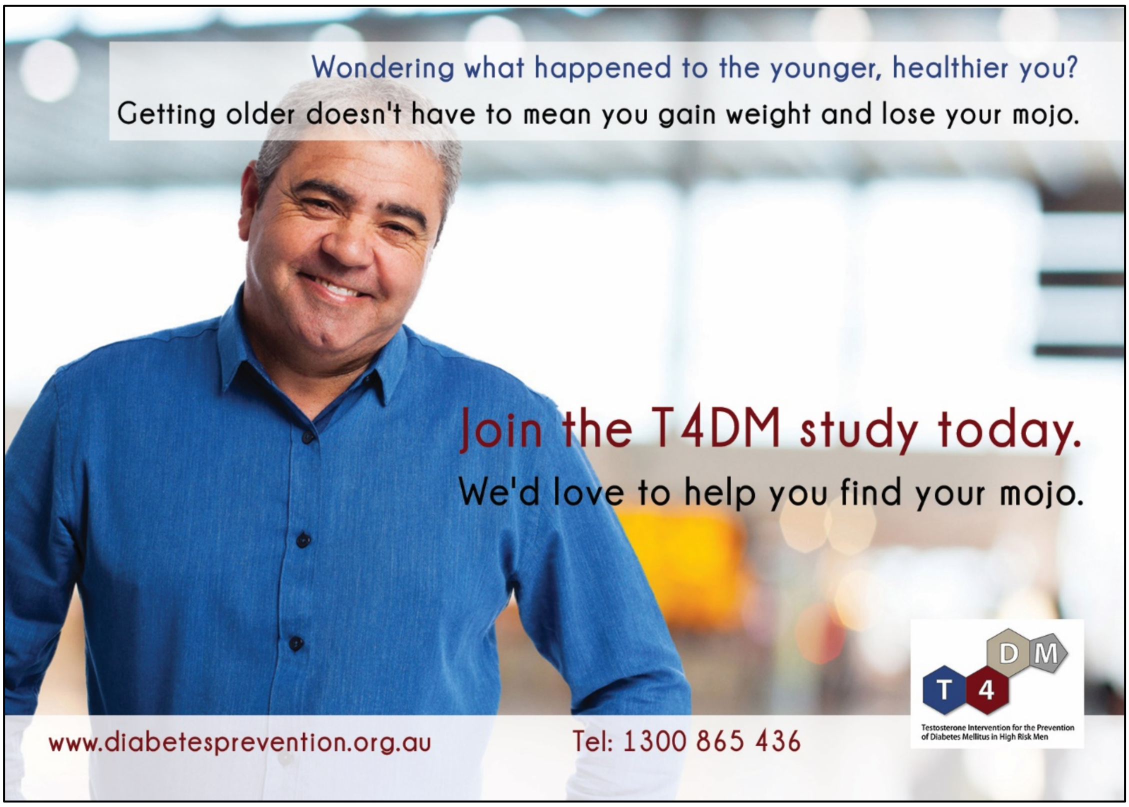


Postcard reverse side

# T4DM Diabetes Prevention Study

**Can testosterone treatment reduce the risk of diabetes in men like you?**

---

Are you male and 50-74 years old? Overweight or obese?
We invite you to join the T4DM Diabetes Prevention Study.

If you join you will receive:

- 2 years of treatment with testosterone or placebo
- 2 years of Weight Watchers membership
- regular support from our dedicated study team

Get started today by completing our quick and easy online questionnaire:

**www.diabetesprevention.org.au**

*For more info or help with the questionnaire*

askt4dm@ctc.usyd.edu.au | 1300 865 436

Proof
