# Supplementary material for: Recruitment of men to a multi-centre diabetes prevention trial: an evaluation of traditional and online promotional strategies
Source: Trials. 2019 Jun 19;20:366. doi: 10.1186/s13063-019-3485-2 (PMC6585027; doi:10.1186/s13063-019-3485-2)
Supplement: Supplementary file 2 — Definitions of common Facebook and Google terms. (PDF 325 kb) [file 13063_2019_3485_MOESM2_ESM.pdf]

Definitions of common Facebook and Google terms

Term	Definition
**Facebook**	
**Facebook Ads Manager**	The online tool provided by Facebook for creation, management and monitoring of Facebook advertising
**Boost**	A Facebook option which allows an advertiser to pay to have a selected Facebook story shown to a larger audience than it would otherwise be shown to. The advertiser can define the characteristics of the audience who should be shown the story.
**Impression**	The number of times that an advertisement or post is shown on screen. Also used in relation to Google advertising.
**Engagement**	A term which encompasses any kind of user interaction with the organisation's Facebook page including clicking on a link, or liking, sharing or commenting on a post.
**Share**	When a user chooses to show content from another Facebook page or website on their own Facebook page
**Split or A/B testing**	Functionality which randomises users to receive different versions of an advertisement or website in order to test which version is the most effective. Available within many online applications including Facebook and Google Advertising.
**Google**	
**Google Adwords**	Google's advertising service. It allows organisations to display advertisements on the Google search results screen when a user searches for keywords from a list defined by the advertiser. Now known as Google Ads.
**Click-through-rate**	The number of times an ad was clicked on, divided by the total number of times the ad was shown. Also known as CTR.
**Maximum cost per click bid**	An amount set by the advertiser as the maximum amount they are willing to pay per click. The actual amount paid may be less than this depending on how much other advertisers have bid. An automated bidding option is available which allows Google to determine the maximum cost per click.
**Cost per click**	The actual amount paid each time a user clicks on an advertisement. Also known as CPC.
**Google Analytics**	Web analytics program which supports website owners to perform real-time monitoring of users' website interactions. For example, it provides statistics on the number of hits and visitors a website receives based on date, time, location and referral source. It also provides metrics on user's behaviour on the website including which pages were visited and how long was spent on each page.
**Goals**	A goal is a website-based desired user action (for example, submitting information on a registration form) which is defined in Google Analytics and tracked. When a user performs the desired action this is called a conversion.
